# Microcirculation in cardiogenic shock supported with extracorporeal membrane oxygenation: the need for a homogeneous population and strict evolution assessment

**DOI:** 10.1186/s13054-018-2214-7

**Published:** 2018-10-29

**Authors:** Santiago Montero, Juliette Chommeloux, Guillaume Franchineau, Alain Combes, Matthieu Schmidt

**Affiliations:** 1 Acute and Intensive Cardiovascular Care Unit, Department of Cardiology, Hospital de la Santa Creu i Sant Pau, Biomedical Research Institute IIB Sant Pau, Universitat Autònoma de Barcelona (UAB), Barcelona, Spain; 2 Assistance Publique-Hôpitaux de Paris, Pitié-Salpêtrière Hospital, Medical Intensive Care Unit, 75651 Paris Cedex 13, France; 3 Sorbonne Universités, Paris 06, INSERM, UMRS_1166-ICAN, Institute of Cardiometabolism and Nutrition, 75651 Paris Cedex 13, France; 4 Service de Réanimation Médicale, iCAN, Institute of Cardiometabolism and Nutrition, Hôpital de la Pitié-Salpêtrière, 47, bd de l’Hôpital, 75651 Paris Cedex 13, France

We read with great interest the article by Yeh et al. about microcirculation in cardiogenic shock supported by venoarterial extracorporeal membrane oxygenation (VA ECMO) [1]. We would like to commend the authors for their efforts to shed some light on this promising field. However, we would like to comment on several points.

The heterogeneity of cardiogenic shock (CS) etiologies and its complex pathophysiology requires the thorough study of selected populations, especially when studying microcirculation. The inflammatory component in CS pathophysiology has become increasingly acknowledged [2] and it may vary significantly depending on its etiology. The authors did not specify the causes of heart failure and, most importantly, half of the patients included in each group were patients who received ECMO for refractory cardiac arrest (E-CPR). Such patients usually present with systemic inflammation response syndrome and thus have important endothelial dysfunction, inflammation, and vasoplegia. In fact, post-cardiac arrest patients have intrinsic impaired sublingual microcirculation [3]. In our opinion, the heterogeneity of the studied population makes it difficult to interpret the outcomes related to microcirculation.

The authors outlined the usefulness of early microcirculatory parameters in predicting 28-day mortality in CS shortly after VA ECMO implantation [1]. They found a well-known lack of relationship between microcirculation and macrocirculation in CS [4]. However, the microcirculatory assessment was performed only after VA ECMO implantation, with no information about the pre-ECMO macro- and microcirculation situation. Thus, we wonder if the worse outcome may also have been due to a profound pre-ECMO microcirculation impairment not sufficiently restored by VA-ECMO despite global hemodynamic normalization. In our opinion, further studies on the microcirculation in CS should specifically assess microcirculation prior to ECMO implantation.

Finally, as almost 50% of CS patients are known to die despite having restored cardiac output [5], it would have been interesting to report the number of non-surviving patients eventually weaned off ECMO and the leading causes of death (multiorgan failure, cerebral anoxia, septic shock, etc.), especially when including such a large population of E-CPR. Along this line, an interesting primary outcome for future studies might be the success of weaning off VA ECMO instead of global 28-day mortality.

## Abbreviations

CS: Cardiogenic shock
ECMO: Extracorporeal membrane oxygenation
E-CPR: ECMO for refractory cardiac arrest
VA ECMO: Venoarterial ECMO
