# Supplementary material for: Microcirculation in cardiogenic shock supported with extracorporeal membrane oxygenation: the need for a homogeneous population and strict evolution assessment
Source: Crit Care. 2018 Oct 29;22:281. doi: 10.1186/s13054-018-2214-7 (PMC6206654; doi:10.1186/s13054-018-2214-7)
Supplement: Related texts

# Reply: Authors’ response

**Authors:** Yu-Chang Yeh, Ya-Jung Cheng, Yih-Sharng Chen

We thank Montero et al. for their comments regarding our article investigating the microcirculation in patients with VA-ECMO life support [1]. First, we agree that the mechanism of microcirculatory dysfunction and its effects on mortality might vary in different primary etiologies of cardiogenic shock; therefore, we mentioned it as the first limitation of our article. Further cooperation in a multicenter trial may help to increase the sample size to answer this question. Second, we agree about the importance of information on the pre-ECMO microcirculation. In daily clinical practice, however, most VA-ECMO placements may occur unexpectedly, in emergency situations, or outside the working hours of research assistants. Further research may be designed to investigate the pre-ECMO microcirculation before elective VA-ECMO placement for cardiogenic shock. Third, the leading causes of death in the 28-day non-survivors were septic shock (29%), postanoxic brain damage (25%), multiorgan failure (25%), and refractory cardiogenic shock (21%). Finally, we agree with Montero et al. that further studies may investigate primary outcomes related to successful weaning from VA-ECMO. We recommend referring to the study of Akin et al. that identified sublingual microcirculation as a novel potential marker for successful weaning from VA-ECMO [6]. It is time-consuming and labor-intensive to investigate microcirculation in VA-ECMO patients, and we need more resources to support VA-ECMO trials. Let us hope that further cooperation in a multicenter trial may help to increase the sample size and power to answer important questions concerning VA-ECMO patients.
